# Infection-Related Declines in Chill Coma Recovery and Negative Geotaxis in *Drosophila melanogaster*

**DOI:** 10.1371/journal.pone.0041907

**Published:** 2012-09-13

**Authors:** Jessica A. Linderman, Moria C. Chambers, Avni S. Gupta, David S. Schneider

**Affiliations:** Department of Microbiology and Immunology, Stanford University, Stanford, California, United States of America; University of California Merced, United States of America

## Abstract

Studies of infection in *Drosophila melanogaster* provide insight into both mechanisms of host resistance and tolerance of pathogens. However, research into the pathways involved in these processes has been limited by the relatively few metrics that can be used to measure sickness and health throughout the course of infection. Here we report measurements of infection-related declines in flies' performance on two different behavioral assays. *D. melanogaster* are slower to recover from a chill-induced coma during infection with either *Listeria monocytogenes* or *Streptococcus pneumoniae*. *L. monocytogenes* infection also impacts flies' performance during a negative geotaxis assay, revealing a decline in their rate of climbing as part of their innate escape response after startle. In addition to providing new measures for assessing health, these assays also suggest pathological consequences of and metabolic shifts that may occur over the course of an infection.

## Introduction

Infection can impact the health of an organism in complex ways. Direct damage to the host caused by pathogenic toxins, damage to host tissue resulting from immune effectors, and the energetic expense of responding to an infection are all known to affect the health of a host [1]–[4]. Surviving an infection requires both resistance, the ability to limit pathogen burden, and tolerance, the ability to minimize the impact a pathogen has on fitness [1], [5], [6]. Understanding the various pathways mediating resistance and tolerance allows for better development of interventions that focus on the maintenance of health throughout an infection. However, dissecting the mechanisms that determine the balance of eliminating pathogens and the damage and energetic cost of mounting that immunological response requires metrics that move beyond survival and allow assessment of health during infection.

Work with the model organism *Drosophila melanogaster* has provided important insights into the roles of different molecular pathways involved in immunity [7]–[9]. *D. melanogaster* provides a genetically tractable system where a large numbers of replicates can be assessed while still being a whole organism system with biological complexity. Additionally, a relationship between infection, immunity, and reproductive fitness in insects has been well established. In environments where nutrients are limited, there is a negative correlation between female fecundity and resistance to bacterial infection in *D. melanogaster* [10]. When *D. melanogaster* are bred in the presence of a microsporidian parasite, the animals have higher fecundity and longer lifespans in the presence of pathogen compared to controls not selected for parasite tolerance and resistance [11]. However, these selected animals are less fit in low nutrient or competitive breeding environments where the pathogen is not present. Additionally, immune challenged females not only have fewer offspring, but those offspring also have shorter lifespans compared to the offspring of unchallenged female *D. melanogaster* [12].

Reproductive fitness is a broad, ecological measure of health. However, in order to determine more direct and causal relationships between an immune response and fitness, a larger panel of metrics assaying different aspects of an animal's ability to respond and react to its environment is needed. Only limited work has been done showing that some physiological metrics used to monitor the health status of humans during infection can also be applied to the fly. Flies infected with *Mycobacterium marinum* undergo a loss of energy stores, a phenomenon similar to the wasting seen in *Mycobacterium tuberculosis* infection in humans [13]. Infection with *Listeria monocytogenes* or *Salmonella typhimurium* results in flies becoming anorexic [14]. *D. melanogaster* larvae infected with *Pseudomonas entomophila* similarly stop eating in this case due to severe gut damage [15], [16]. Infection with *Listeria monocytogenes* or *Streptococcus pneumoniae* results in a loss circadian rhythms in flies [17]. One of the limitations of these assays is that wasting, anorexia, and sleeplessness are intricately linked to the immune response and form feedback loops as they can have negative or positive effects on the ability of *D. melanogaster* to survive an infection [13], [14], [17]. Additional assays would allow for the monitoring of an animal's health throughout infection, potentially providing a more complete picture throughout the course of infection and allowing for assessment of recovery after a non-lethal infection.

Here we report on assays that are not measures of natural declines in infection, but rather measure the flies' ability to recover from stress or react to stimuli. Such assays are commonly used in the field of aging research in *D. melanogaster*, which has long recognized that measures of both healthspan and longevity are critical to understanding the biology of aging. We hypothesized that these metrics could also be used to assess health during infection because *D. melanogaster* shows an age-related up-regulation of inflammatory genes and expression patterns that characterize aging and the induction of an immune response in these animals are related [18]. Additionally, the wasting and loss of circadian rhythms reported to occur during infection in flies are seen in aged flies as well [19], [20].

**Figure 1:**
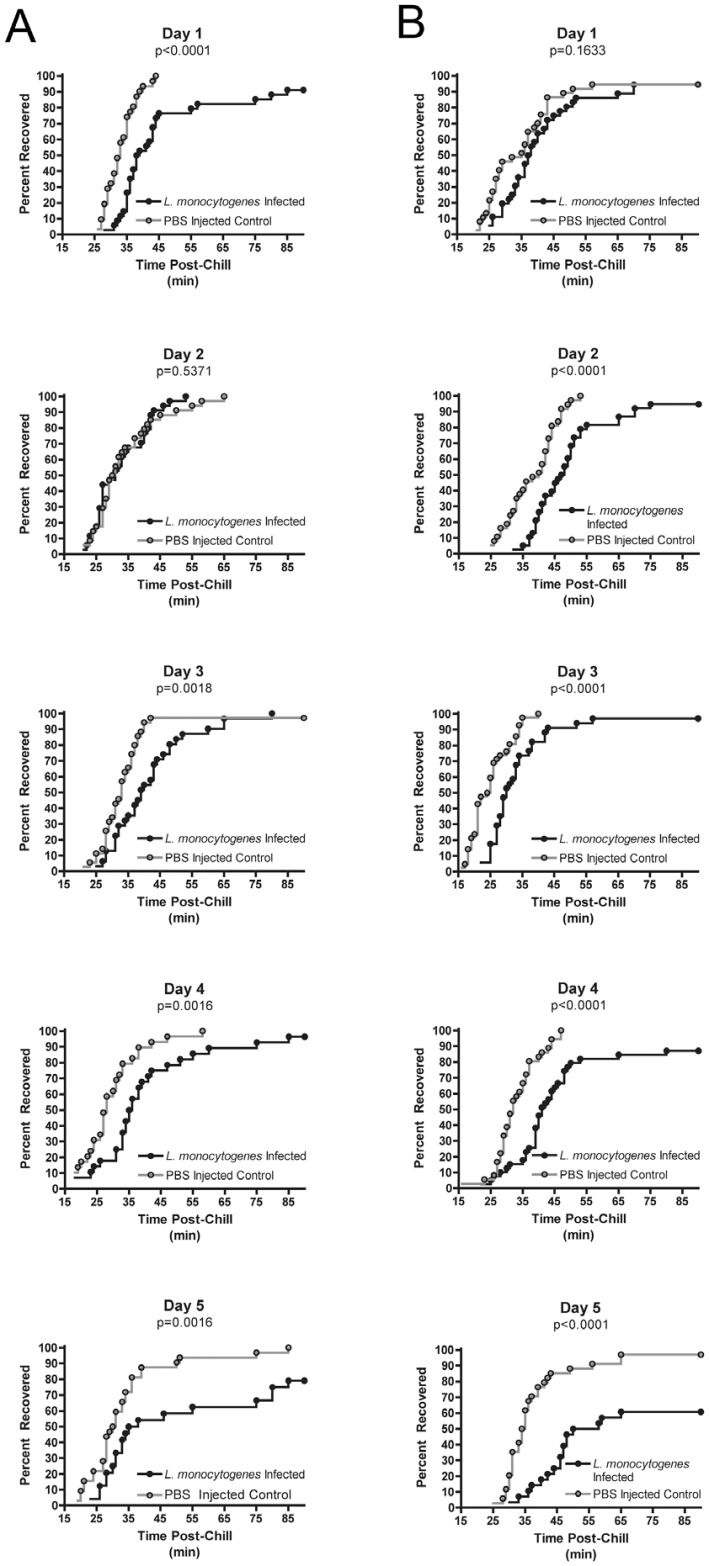
*L. monocytogenes* infection impairs chill coma recovery. Representative chill coma recovery graphs for Days 1–5 post- *L. monocytogenes* infection for (A) Oregon-R and (B) w1118, The y-axis indicates the percentage of flies that have recovered from chill coma such that they have stood by the time indicated in minutes on the x-axis.

Here we show infection-related deficits for two behavioral assays – chill coma recovery and negative geotaxis. When insects are exposed to low temperatures, they enter into a reversible period of immobility referred to as chill coma. While the physiological causes behind induction and recovery from this coma are incompletely understood, the amount of time it takes *D. melanogaster* to be able to stand after returning to a warmer temperature is altered by the animal'ss environment prior to cold exposure, its energy stores, and its age [21]–[24]. Negative geotaxis is the measure of how quickly a fly is able to climb vertically after being tapped to the bottom of a vessel as part of its innate escape response. Negative geotaxis is measured by either the distance an animal is able to climb in a set time or the length of time it takes an animal to climb a set distance. Negative geotactic ability has been shown to be sensitive to oxidative stress, age, and previous cold exposure, but not to fungal infection [25]–[29]. We hypothesize that the physiological changes that cause performance deficits in both of these assays are affected by infection, such that both assays can be used to detect decreases in health during infection.

**Figure 2:**
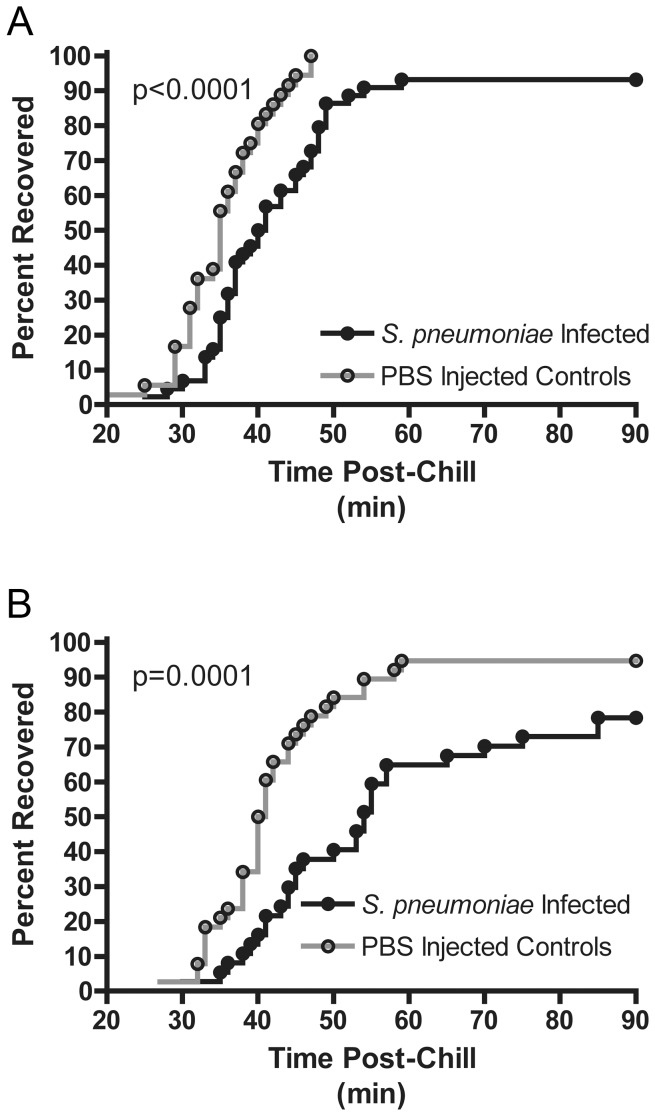
*S. pneumoniae* infection impairs chill coma recovery. Representative chill coma recovery graphs for *S. pneumoniae* infected (A) Oregon-R and (B) w1118. The y-axis indicates the percentage of flies that have recovered from chill coma such that they have stood by the time indicated in minutes on the x-axis. There is a significant increase in chill recovery time for infected flies compared to their PBS injected controls.

## Materials and Methods

### *D. melanogaster* and Bacterial Strains

Work was done using the Oregon-R and w1118 (Bloomington 6326) strains of *Drosophila melanogaster.* All flies were bred in round (6 oz) polypropylene fly bottles (Genesee Scientific) where 10 males and 10 females were placed together for forty-eight hours and then removed. Twenty-four hours after eclosion, flies were briefly anesthetized with CO_2_ for a period of no more than five minutes, were separated according to sex, and placed in narrow polystyrene ﬂy vials (25×95 mm, Genesee Scientific) containing 20 flies per vial. Each vial contained dextrose medium containing 129.4 g dextrose, 7.4 g agar, 61.2 g corn meal, 32.4 g yeast, and 2.7 g tegosept per 1L of cooked food [14]. Infections were performed with *Listeria monocytogenes* strain 10403 S or *Streptococcus pneumoniae* strain SP1. *S. pneumoniae* was frozen at OD_600_ 0.11 in 10% glycerol, diluted three-fold in Brain-Heart Infusion (BHI) Broth upon thawing, incubated 3 hour at 29°C, and concentrated by microfuge. Bacteria were diluted in PBS to an OD_600_ corresponding with 10,000 bacteria per 50 nL for Oregon-R flies 2,000 bacteria per 50 nL for w1118. Sterile PBS was used for control injections. *L. monocytogenes* was grown from stock overnight in BHI broth and diluted in PBS to 1000 bacteria per 50 nL. BHI diluted with equivalent amounts of PBS was used into controls.

**Figure 3:**
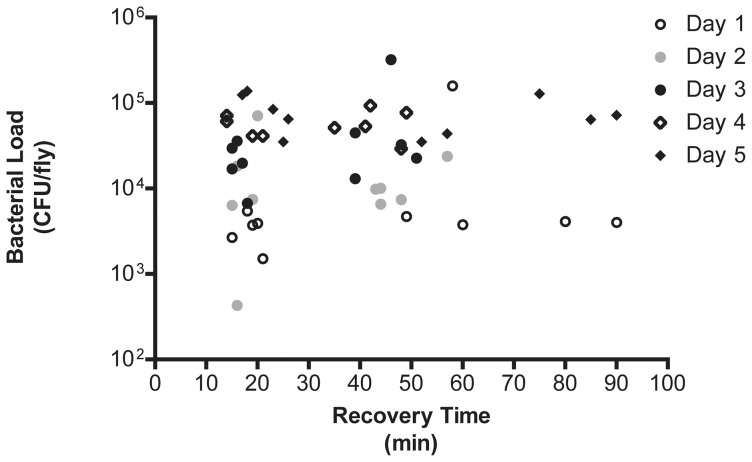
Chill coma recovery time does not correlate with pathogen load for *L. monocytogenes*-infected Oregon-R. Bacterial load was calculated for the five quickest and slowest flies to recover each day throughout the course of infection. There is no statistical correlation between the two (r^2^ = 0.0243).

### Injections and Bacterial Load Measures

Males reared at 25°C and 65% humidity in a 12 h light:dark cycle for five to seven days post-eclosion were used for injection. Flies were anesthetized using CO_2_ for no more than 5 minutes, and injections were done with a pulled glass capillary needle and a picospritzer used to inject 50 nL of liquid per fly. Injection volume was calibrated by measuring the expelled drop in oil. Bacterial load at the time of injection and throughout infection was monitored by using a Spiral Biotech Autoplate Spiral Plater (Norwood, MA) system to plate individually homogenized flies on LB Agar plates for *L. monocytogenes* and blood agar plates for *S. pneumonia*. After overnight growth, colonies were counted and colony forming unit concentration calculated using Spiral Biotech Qcount (Norwood, MA). All flies were returned to rearing vials containing 20 flies after injection, moved to an incubator kept at 29°C, and were given at least 24 hours to recover before any behavioral assays were performed. Careful attention was paid to injection volumes, time on CO_2_ and recovery periods being equivalent for both infected and PBS injected control animals as such stresses could affect performance on behavioral assays [30]–[32].

**Figure 4:**
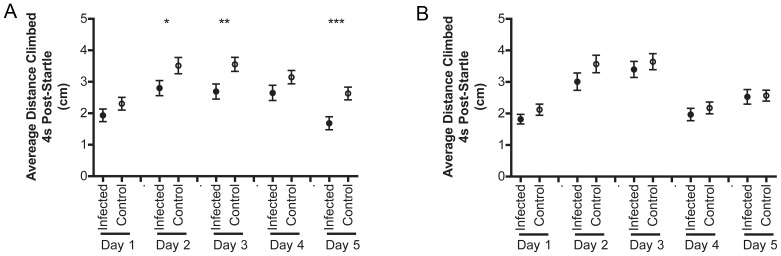
Oregon-R, but not w1118, flies show *L. monocytogenes* infection-related deficits in negative geotaxis. Mean and standard error of distance climbed by 4 s post-startle for a representative trial of *L. monocytogenes* infected (A) Oregon-R and (B) w1118 over the course of infection. Only Oregon-R flies show a significant decline in climbing with infection (*p<0.05, **p<0.01, ***p<0.001).

### Chill Coma Recovery

Each day a new group of infected and control flies were transferred to clean glass vials (20 flies per vial) without anesthesia around noon and placed in melting ice in a bucket for three hours to induce chill coma. Prior to beginning the assay, flies were moved from the vials to individual wells of a 48 well plate while being kept on ice. A timer was started once the plates were moved from the ice to a bench at room temperature and flies were considered recovered when they were able to stand. Each fly was checked by eye for recovery once a minute for the first 60 minutes, and every five minutes for the remaining 90-minute period. Data is expressed as the percentage of flies that have stood by each time point. A single set of flies was not used throughout the course of infection due to the reported effect of exposure to cold on longevity and immunity [33]–[35]. Six or seven sets of 40 infected flies and 40 controls were injected at the same time and a new set was tested each day until 50% of the infected flies had died, the first five days post-infection for *L. monocytogenes* experiments and the first day post*-S. pneumoniae* infection. The recovery curves for infected animals were compared to control injected flies for each genetic background, bacterial infection, and day post-infection separately using a Logrank test in Prism (GraphPad). Three replicates were done for each condition, and an overall p value was calculated using Fisher's combined probability test in R.

### Negative Geotaxis

The negative geotaxis assay was performed in a manner similar to that published by Gargano et al [36]. 80 infected and 80 control flies were transferred once a day in the early afternoon without anesthesia into 15 cm tall clean glass vials, with the same 20 flies who were reared together being placed in a glass tube to perform the assay together. The glass vials were placed in a rectangular frame to keep them upright and put in front of a light box at room temperature in a chamber kept between 50% and 60% humidity. After a ten-minute acclimation period, the frame was tapped three times and climbing was captured with time-lapse photographs taken once per second post startle. Flies were then transferred without anesthesia back into their rearing vial. The same set of flies was tested each day throughout the course of the experiment until 50% of the infected flies had died, five days for *L. monocytogenes* infection and one day for S. *pneumoniae* infection. ImageJ particle analysis was then used to determine the height animals had climbed 4 s post-startle. The distance climbed by 4 seconds post startle was compared between infected and control injected groups using a two way ANOVA in R for *L. monocytogenes*-infected animals or Mann-Whitney test in Prism (GraphPad) for S. *pneumoniae*-infected flies. Three replicates were done for each condition, and an overall p value was calculated using Fisher's combined probability test in R.

## Results

### *L. monocytogenes* and *S. pneumoniae* infection impairs chill coma recovery

Experiments were done with two infections and two strains of *D. melanogaster* that have previously been reported to undergo infection-related changes in circadian rhythms, display sickness-induced anorexia, and have revealed different infection-dependent and background-dependent resistance and tolerance mechanism [14,17, unpublished observation]. Infection with *L. monocytogenes* was lethal for both Oregon-R and w1118 flies with a median time to death of 6 days (Figure S1A and B.) PBS injected controls had a median time to death greater than 30 days for Oregon-R and w1118. Flies were infected with *S. pneumoniae* at doses titrated such that the majority of flies were dead two days post-infection (Figure S1C and D). Greater than 80% of S. *pneumoniae*-infected flies died within the first three days after infection, but a small percentage were able to clear the bacteria and survive greater than 30 days.

To measure chill coma recovery, flies were placed on ice for three hours to induce a chill coma. Flies were then returned to room temperature and monitored for recovery as determined by their ability to stand. Both Oregon-R and w1118 flies showed a delay in time to recovery during infection with either *L. monocytogenes* (Figure 1) or *S. pneumoniae* (Figure 2). *L. monocytogenes* infected Oregon-R flies showed a significant delay in recovery compared to PBS-injected controls on days 1, 2, 4, and 5 post-infection when all three replicates were combined, but the difference did not reach significance each day for each individual trial (Figure 1A; Logrank test and Fisher's combined probability test – Day 1: p = 0.0007, Day 2: p = 0.0147, Day 3:p = 0.2506, Day 4: p<0.0001, Day 5: p<0.0001). ) *L. monocytogenes*-infected w1118 similarly showed a significant delay in recovery from chill coma for infected flies compared to PBS-injected controls throughout the course of infection as determined by Logrank and Fisher's combined probability test (Figure 1B; Day 1: p = 0.002, Day 2–5: p<0.0001). Each trial did reach individual significance on Days 2–5. , Both Oregon-R and w1118 flies also showed *S. pneumoniae*-related increases in chill coma recovery times compared to controls for each trial (Figure 2; Oregon-R: p<0.0001; w1118: p<0.0001). While numbers were not great enough to do rigorous statistical analysis of survival, most flies did recover from chill coma but a notable number of w1118 flies infected with *S. pneumoniae* would not respond to gentle shaking even after 90 minutes at room temperature and were presumed dead (Tables S1 and S2).

We examined whether there was a relationship between bacterial load and recovery time for *L. monocytogenes*-infected Oregon-R flies. Bacterial loads were measured for the five fastest and five slowest flies to recover each day, but no relationship was found (Figure 3, p = 0.3431).

### Oregon-R, but not w1118, show *L. monocytogenes* infection-related deficits in negative geotaxis

*L. monocytogenes*-infected Oregon-R flies showed declines in the height climbed at 4 s post-startle during infection. Comparison of the distance climbed by infected versus uninfected flies showed significant differences (Figure 4A; Trials: p<0.0001, p = 0.0021, p<0.0001 Combined: p<0.0001). No differences were seen in w1118 flies infected with *L. monocytogenes* (Figure 4B) or in either background during *S. pneumoniae* infection (data not shown).

## Discussion

Here we demonstrate a significant impairment in chill coma recovery during *Listeria monocytogenes* or *Streptococcus pneumoniae* infection. Chill coma recovery is a process that is known to have both genetic and environmental influences [37]. As we did not see a correlation between bacterial load and chill coma recovery time, we hypothesize that direct damage to the host inflicted by the bacteria is not responsible for the increase in recovery time during infection. Rather, we hypothesize that it is a more complicated interaction that could be dependent on tissue damage due to the immune response or the energetic expenses of an immune response.

It has been shown in *D. melanogaster* and other insects that expression of immune response genes are increased in response to long term or repeated exposure to cold suggesting potential shared responses to cold and infection [38], [39]. However, a single exposure to chill as performed here did not show an increase in genes considered part of the immune response [38], [40]. A single chill period resulted in up-regulation of more general stress response genes, including genes that are also up-regulated during infection. The up-regulation of heat shock proteins, particularly Hsp70, is known to be of functional importance in response to both stresses [41]–[43], suggesting some shared protective mechanisms. The gene Frost likewise plays a role in chill coma recovery [44] and is known to be up-regulated during infection [45]. These may highlight the importance of protection against cellular damage to survival of each of these stresses. The combined impact of chill and infections may overwhelm the protective mechanisms of these flies, resulting in delayed chill coma recovery or even damage resulting in death.

It is also likely that relationship between the physiology of infection and delayed chill coma recovery is due to the metabolic changes that occur during infection. Mounting an immune response is known to have an energetic cost, hypothesized to result in trade offs between immunity and response to other stresses [3], [46], [47]. Insufficient energy stores could contribute to the flies increase in chill coma recovery time during infection. Flies infected with *Mycobacterium marinum* exhibit a form of wasting that results in reduced glycogen and triglyceride stores [13]. *D. melanogaster* lines selected to better survive cold had overall higher energy stores and higher levels of glycogen and protein [24]. However, the relationship between energy stores and chill coma recovery time is not that straightforward. For example, insulin-like peptide deficient flies have higher fat stores and yet show impaired chill recovery [48]. It is likely that both sufficient energy stores and expression of proteins, such as insulin, that allow for quick access to those stores are important. Further investigation into the changes of metabolic stores and regulation of those stores in infected flies may suggest a mechanisms underlying delayed chill coma recovery during infection.

We also show a decline in negative geotactic ability, as measured by how high a fly climbs 4 s post startle, in *L. monocytogenes* infected Oregon-R flies. The benefit of a negative geotaxis assay is the possibility of being able to follow the same set of flies throughout the course of an infection. While cold hardening has been reported to affect lifespan and immunity [33]–[35], we found no indication that daily tests of negative geotaxis altered survival, had an impact on bacterial load, or affected results in future tests of climbing (data not shown). Unfortunately, this assay showed less robust infection-related changes than chill coma recovery. Despite this fact, there may still be interesting biological insights to be gained from further study of this phenomenon.

Here we present measures of both chill coma recovery and negative geotaxis could be useful in understanding the time course and degree to which different infections alter health in different genetic backgrounds of *D. melanogaster*. Previous work examining the relationship between pathogen load and survival suggest complex interactions between health, pathogen load, and immunity. Measures of health using these non-lethal behavioral assays may provide further insight into the mechanisms by which this organism is able to maintain health in the face of infection.

## Supporting Information

Figure S1 Survival curves for infected and control flies. Representative survival curves for (A) *L. monocytogenes* infected and PBS injected control Oregon-R flies, (B) *L. monocytogenes* infected and PBS injected control w118 flies, (C) *S. pneumoniae* infected and PBS injected control Oregon-R flies and *(D) S. pneumoniae* infected and PBS injected control w1118 flies.
(TIF)

Table S1 Fraction of *L. monocytogenes* infected and control flies not responding to tactile stimulation 90 minutes post-chill. The number of flies challenged with PBS or *L.monocytogenes* that did not respond to tactile stimulation following 90 minutes post-chill incubation is recorded.
(XLSX)

Table S2 Fraction of *S. pneumoniae* infected and control flies not responding to tactile stimulation 90 minutes post-chill. The number of flies challenged with *S.pneumoniae* that did not respond to tactile stimulation following 90 minutes post-chill incubation is recorded.
(XLSX)
