# Supplementary figures and images for: Infection-Related Declines in Chill Coma Recovery and Negative Geotaxis in Drosophila melanogaster
Source: PLoS One. 2012 Sep 13;7(9):e41907. doi: 10.1371/journal.pone.0041907 (PMC3441536; doi:10.1371/journal.pone.0041907)

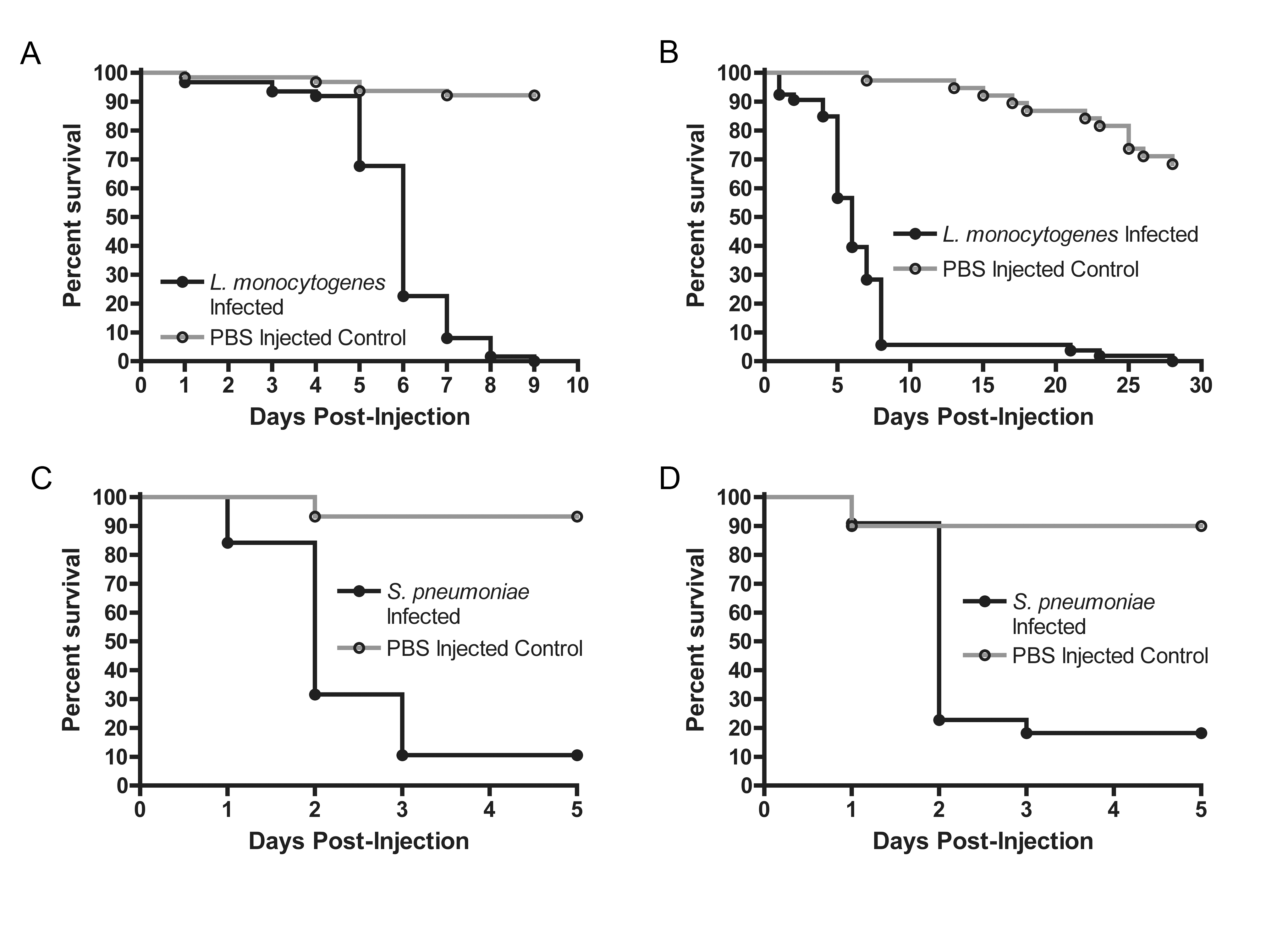

Supplement: Figure S1 — Survival curves for infected and control flies. Representative survival curves for (A) L. monocytogenes infected and PBS injected control Oregon-R flies, (B) L. monocytogenes infected and PBS injected control w118 flies, (C) S. pneumoniae infected and PBS injected control Oregon-R flies and (D) S. pneumoniae infected and PBS injected control w1118 flies. (TIF) [file pone.0041907.s001.tif]
